# Three-Dimensional Nuclear Telomere Profiling as a Biomarker for Recurrence in Oligodendrogliomas: A Pilot Study

**DOI:** 10.3390/ijms21228539

**Published:** 2020-11-12

**Authors:** Macoura Gadji, Shubha Mathur, Brigitte Bélanger, Jaganmohan Reddy Jangamreddy, Josée Lamoureux, Ana Maria Crous Tsanaclis, David Fortin, Régen Drouin, Sabine Mai

**Affiliations:** 1 Division of Genetics, Department of Pediatrics, Faculty of Medicine and Health Sciences, University of Sherbrooke, 3001 12th Avenue North, Sherbrooke, QC J1H 5N4, Canada or macoura.gadji@ucad.edu.sn (M.G.); Brigitte.Belanger@USherbrooke.ca (B.B.); Josee.Lamoureux@USherbrooke.ca (J.L.); regen.drouin@crchudequebec.ulaval.ca (R.D.); 2 Departments of Physiology and Pathophysiology, Rady Faculty of Health Sciences, Cell Biology, Research Institute of Oncology and Hematology (RIOH), CancerCare Manitoba (CCMB), The Genomic Centre for Cancer Research and Diagnosis (GCCRD), The University of Manitoba, 675 McDermot Avenue, Winnipeg, MB R3E 0V9, Canada; s_mathur3110@hotmail.com; 3 Faculty of Medicine, Pharmacy and Odonto-Stomatology (FMPO), National Centre of Blood Transfusion of Dakar (CNTS), The Cheikh Anta Diop University of Dakar (UCAD), Dakar Fann BP 5002 Pt E, Senegal; 4 UR Advanced Therapeutics Pvt Ltd., ASPIRE-BioNEST, School of Life Sciences, University of Hyderabad, Hyderabad 500046, India; jjmreddy@gmail.com; 5 Department of Pathology and cellular Biology, Faculty of Medicine, University of Montreal, Montreal, QC H3C 3J7, Canada; ana.maria.crous.tsanaclis.chum@ssss.gouv.qc.ca or; 6 Division of Neurosurgery, Department of Surgery, Faculty of Medicine and Health Sciences, University of Sherbrooke, Sherbrooke, QC J1H 5N4, Canada; David.Fortin@Usherbrooke.ca; 7 Division of Medical Genetics, Department of Pediatrics, CHU de Quebec – ULaval, Faculty of Medicine, University Laval, Quebec City, Quebec, QC G1V 4G2, Canada

**Keywords:** oligodendroglioma, gliomas, telomere, three-dimensional, nuclear organization

## Abstract

Mechanisms of recurrence in oligodendrogliomas are poorly understood. Recurrence might be driven by telomere dysfunction-mediated genomic instability. In a pilot study, we investigated ten patients with oligodendrogliomas at the time of diagnosis (first surgery) and after recurrence (second surgery) using three-dimensional nuclear telomere analysis performed with quantitative software TeloView^®^ (Telo Genomics Corp, Toronto, Ontario, Canada). 1p/19q deletion status of each patient was determined by fluorescent in situ hybridization on touch preparation slides. We found that a very specific 3D telomeric profile was associated with two pathways of recurrence in oligodendrogliomas independent of their 1p/19q status: a first group of 8 patients displayed significantly different 3D telomere profiles between both surgeries (*p <* 0.0001). Their recurrence happened at a mean of 231.375 ± 117.42 days and a median time to progression (TTP) of 239 days, a period defined as short-term recurrence; and a second group of three patients displayed identical 3D telomere profiles between both surgery samples (*p* > 0.05). Their recurrence happened at a mean of 960.666 ± 86.19 days and a median TTP of 930 days, a period defined as long-term recurrence. Our results suggest a potential link between nuclear telomere architecture and telomere dysfunction with time to recurrence in oligodendrogliomas, independently of the 1p/19q status.

## 1. Introduction

Oligodendrogliomas are one of different entities of diffusely infiltrating malignant gliomas, which are the most common primary brain tumors in adults. Indeed, gliomas are composed of oligodendrogliomas [World Health Organization (WHO) grades II and III], diffuse or circumscribed astrocytomas (WHO grades II, III and IV or glioblastoma), ependymomas, and other rare histologic groups [1,2,3,4,5,6]. Gliomas account for 40% of primary brain tumors [5], while tumors of the brain and the central nervous system represent the 17th most common cancer type, with an estimated 297,000 new cases worldwide [7,8,9]. Oligodendrogliomas are characterized by uniform, round to oval nuclei with crisp nuclear borders, delicate speckled chromatin, and (in formalin fixed tissue) perinuclear cytoplasmic clearing, with a background of delicate branching small vessels [10,11,12]. Thus, the triad of uniformly round nuclei, perinuclear haloes, and an even cellular distribution together with a delicate vascular web (the so-called chicken-wire pattern) in a context of infiltration of adjacent brain parenchyma, represents the classical criteria for histopathological diagnosis of oligodendrogliomas [3,13]. Oligodendrogliomas are subdivided in two histological subtypes as low grade oligodendroglioma (WHO grade II) displaying high cellularity, cytologic atypia, necrosis, vascular proliferation, and anaplastic oligodendroglioma (WHO grade III) showing significant mitotic activity with a minimum of “conspicuous microvascular proliferation and/or brisk mitotic activity” important for diagnosis [2,3,4]. In summary, histopathological assessment of oligodendroglial tumors remains the gold standard to establish a diagnosis in everyday practice [3,13].

However, histopathological delineation of diffuse gliomas can be difficult because of vague and subjective histopathological criteria [2,3,14], mostly in intermingled oligodendroglial cells and astrocytic cells. In a significant number of these lesions, the microscopic morphology is not so clear-cut, and the distinction from other glial lesions may be difficult [15]. Identification of characteristics inherent to the tumor type is further complicated by regional tumor heterogeneity and sampling error. One consequence of this subjectivity in diagnostic criteria is a high level of intraobserver and interobserver variability, which decreases reproducibility [16,17,18,19]. To face these objective limitations, molecular studies highlighted important breakthroughs in gliomagenesis. Indeed, the first and most important biomarker was the 1p/19q codeletion (1p-/19q-) that is a predictive and prognostic biomarker of oligodendrogliomas [3,17,20]. This 1p-/19q- genetic signature is also recognized as a diagnostic biomarker of pure oligodendrogliomas [3,14,18], which is reinforced by the discovery of the translocation between chromosomes 1 and 19 as the chromosomal mechanism generating these deletions [3,18,20,21,22]. It has been reported that in classic oligodendrogliomas, the 1p/19q tumor status is a powerful predictor of patient survival, even after recurrence [20]. These cytogenetic and molecular findings have led to the WHO 2016 classification in which oligodendrogliomas have a strict molecular definition displaying an *IDH* (Isocitrate dehydrogenase) alteration and evidence for deletion of both 1p and 19q for an integrated diagnosis [4,23,24,25]. However, oligodendrogliomas are unfortunately considered incurable and invariably recur, despite occasionally observed impressive long-term responses to chemotherapy. Mechanisms of recurrence are poorly understood in oligodendrogliomas.

In this context, different potential biomarkers for diffuse gliomas have been proposed, such as: *TERT* (telomerase reverse transcriptase) promoter mutations, amplification/mutations in *EGFR* (epidermal growth factor receptor) gene, mutations/deletions in *PTEN* (phosphatase and tensin homologue) and *MGMT* (O-6-methylguanine-DNA methyltransferase) promoter methylation. *TERT* promoter mutations are present in a high percentage of gliomas (80–90%) [26]. Indeed, two genetic variants located near the telomerase genes *TERC* and *TERT* are associated both with increased risk of high-grade glioma and with longer telomere length [27]. The presence of frequent mutations in telomerase genes in gliomas points to an important role for telomere biology in glioma development [28]. The 3D organization of the genome and nucleus are essential components of tumorigenesis as stipulated by Theodore Boveri (1862–1915) a century ago [29,30,31]. Telomeres, the nucleoprotein complexes located at the end of eukaryotic chromosomes, have essential roles in preserving chromosomal integrity. Intact telomeres prevent terminal fusions, degradation of the chromosome ends, and contribute to the adequate chromosome positioning within the nucleus [32]. Telomeres consist of a tandem repeated DNA sequence (TTAGGG_(n)_ in vertebrates) that varies in length from 5 to 15 kb in humans [33,34]. Telomere dysfunction and/or erosion is known to promote chromosomal instability (CIN) and carcinogenesis [35,36,37]. In most human somatic cells, telomeres act as a mitotic clock that limits cell division [38]. Telomeres are organized in a very particular way within the 3D space of the nucleus, where in normal cells, they do not overlap [39] and are localized in microterritories [40]. Telomeres of tumor cell nuclei however, show an altered 3D nuclear organization and form telomere aggregates (TAs) that can be observed in the interphase nucleus [39]. Previously, we described a new stratification tool based on 3D telomeric architecture using a series of adult glioblastomas (GBMs) [41].

In order to understand the cellular mechanisms governing recurrence in oligodendroglial tumors, we characterized in the present pilot study the nuclear telomeric architecture of oligodendroglial nuclei before (at diagnosis/first surgery) and after recurrence (second surgery). We found two groups of patients dichotomized by their 3D telomere profiles at diagnosis and at recurrence with either oligodendrogliomas that correspond to short- and long-term recurrence. We conclude that 3D telomere profiles may predict pathways of recurrence and disease progression in oligodendrogliomas. Furthermore, the degree of telomere dysfunction might be linked to the time of recurrence in oligodendrogliomas.

## 2. Results

### 2.1. Clinical Surrogates and Pathological Diagnosis

The patients included in this study consist of 10 individuals, four men and six women ranging in age from 29 to 79 years (mean: 44.8 ± 14.077 years; median age: 41 years). The patients all depicted a pre-operative presumptive diagnosis of glioma that was confirmed as oligodendroglial at surgery (Table 1) according to WHO criteria [2]. The patients presented with variable clinical findings, depending on the location of their tumors. The common tread for these 10 patients is that they required reoperation for symptomatic progression, allowing us to acquire a second surgical sample. Only patient P4, had four surgeries due to disease progression, which provided us four samples to analyze for that case. Indeed, patient 4 progressed from indolent oligodendroglioma (first surgery) to an oligodendroglioma (second surgery) termed as patient P4a also from oligodendroglioma (third surgery) to oligoastrocytoma (fourth surgery) named as patient P4b; allowing to consider 11 patients in this study.

At our institution, oligodendroglial tumors are now treated according to the following paradigm: the patients are first submitted to surgery for diagnostic and cytoreductive purposes, and the most extensive resection applicable is performed to maximize the extent of resection. Low-grade tumors are then observed until progression, whereas high-grade tumors are treated with upfront chemotherapy, when 1p/19q deleted; individual decision based on the tumor board consensus is used for non-deleted patients. Upon progression, most patients are re-operated, and a second line of treatment (radiotherapy, or a second line of chemotherapy) is initiated.

The patients of this study were initially operated. Three patients were then exposed to radiation therapy upfront after surgery; two of them went to chemotherapy whereas the remaining seven patients were followed until clinical or radiological signs of progression. Upon progression, two were treated with radiation therapy whereas five were exposed to chemotherapy. All but two patients underwent a second line of treatment after having failed the first line, and seven patients were exposed to three or more line of treatment. Overall, all patients were exposed to at least one line of chemotherapy, and all were exposed to radiation therapy.

### 2.2. Molecular Cytogenetic Analysis: FISH for 1p/19q

**Figure 1:**
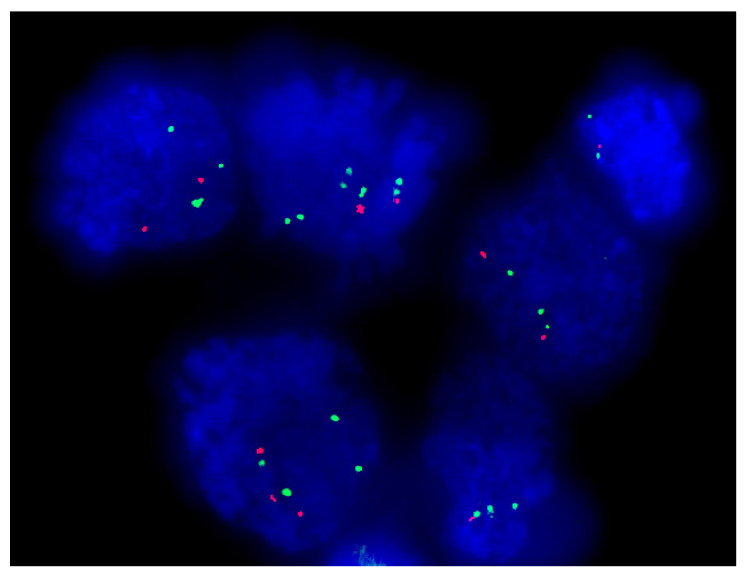
Example of 1p/19q FISH results from patient P5 showing an imbalance between number of copies of chromosome 1p on cells in touch preparation slides (1x 1000 magnification). FISH performed with the LSI 1p36 Spectrum Orange probe (red signals) and the control part LSI 1q25 Spectrum Green probe (green signals).

The FISH technique for 1p/19q deletion was performed successfully on 22 touch preparation (TP) smear slides from 10 patients (one slide for each surgery per patient and two more TP smear slides for patient P4 for a third and fourth surgery). FISH analyses of the data were performed following our already published work [3]; briefly an average of 200 nuclei per slide were scored in the FISH analysis. For each cell, the ratio between the paired probes on chromosomes 1 and 19 was analyzed. The green signal in the used probes served as a control, and the deletion per nucleus showed that the number of green signals was higher than the number of red signals. A case was considered deleted with 1p, 19q, or both when the scored nuclei displayed an imbalance between green and red signals The results displayed four patients at both respective surgeries with 1p-/19q- (P1, P2, P6, and P7 i.e., 8 TP slides), five patients at both respective surgeries (P4a first and second surgeries, P5, P8, P9, and P10 i.e., 10 TP slides) with 1p-/19q- or 1p- or 19q- in a polysomic status (i.e., aneuploid tumors in which there is more than two copies for the green (control signals) and for the red signals (target signals) but with an imbalance between both signals in a ratio as 2/3, 2/4, 2/5, 3/4, 3/5, 4/5…) [3] (Figure 1; Table 1). One patient at both respective surgeries (P3 i.e., 2 TP slides), displayed 1p- alone and one patient (P4b third and fourth surgeries i.e., 2 TP slides) displayed 19q- alone (Figure 1; Table 1).

These results suggest that 1p-/19q-, despite being a solid biomarker of therapeutic response of oligodendrogliomas [3,20] is unable to predict recurrence in these diseases (Figure 1; Table 1).

### 2.3. Three-Dimensional Nuclear Telomere Architecture Analyses

**Figure 2:**
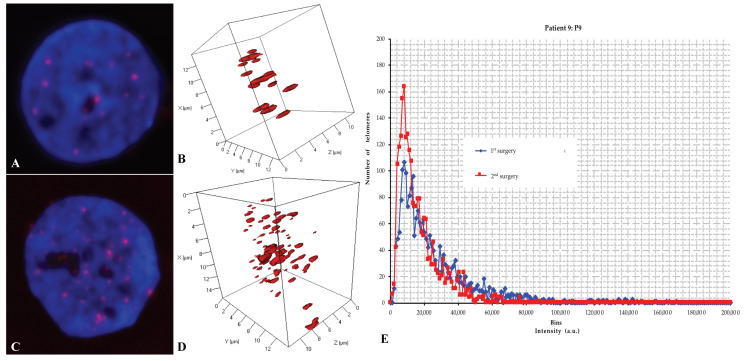
In an example of a group-1 patient (from patient 9: P9), a representative nucleus (1 × 1000 magnification) with 2D QFISH at first surgery (**A**) and at second surgery (**C**); representative nucleus with 3D QFISH at first surgery (**B**) and at second surgery (**D**); and a representative combined 3D telomere profiles displaying the distribution of the total number of signals (total number of telomeres) versus their intensities (telomere length) at both surgeries, respectively (**E**) for patients with short-term recurrence (P9: TTP = 232 days and OS = 1103 days).

**Figure 3:**
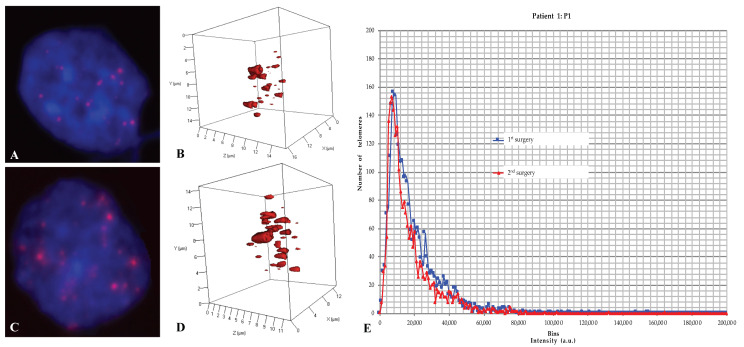
In an example of a group-2 patient (from patient 1: P1), a representative nucleus (1 × 1000 magnification) with 2D QFISH at first surgery (**A**) and at second surgery (**C**); representative nucleus with 3D QFISH at first surgery (**B**) and at second surgery (**D**); and a representative combined 3D telomere profiles displaying the distribution of the total number of signals (total number of telomeres) versus their intensities (telomere length) at both surgeries, respectively (**E**) for patients with long-term recurrence (P1: TTP = 894 days and OS = 5212 days).

All samples were analyzed in a blinded fashion. We first analyzed the total number of telomeres versus their length and number of aggregates in each cell of each sample using TeloView^®^ [41,42,43,44] (Figure 2, Figure 3 and Figures S1–S8, Table 2 and Table S1). Subsequently, the 3D telomere profile was defined by the distribution pattern of telomeres per sample according to their intensity (length and aggregate formation) (Figure 2, Figure 3 and Figures S1–S8).

According to the 3D telomere profiles at first and second surgery, and blinded to the clinical data, our analysis allowed us to classify all the patients into two groups dichotomized by two different genotypic modes of recurrence of the tumor:

Group 1 (P2, P3, P4b, P5, P7, P8, P9 and P10): These patients displayed significantly different 3D telomere profiles between both surgeries (*p* < 0.0001) (Figure 2, Table 2 and Table S1)

Group 2 (P1, P4a, and P6): These patients displayed identical 3D telomere profiles between both surgeries (*p* > 0.05) (Figure 3, Table 2 and Table S1).

### 2.4. Comparative Analyses of the 3D Telomere Profile between the Two Groups

We compared all 3D nuclear telomere parameters (means ± standard deviation per cell) between the first and second surgery in each group-1 patient and in each group-2 patient.

#### 2.4.1. The Number of Signals, i.e., the Number of Telomeres

We analyzed the number of signals i.e., the number of telomeres at first surgery and second surgery. We found an increasing number of telomeres in the second surgery for both groups. However, this difference was significantly higher for group-1 patients (*p* < 0.0001) whereas it was not as significant for group-2 patients (*p* = 0.036).

#### 2.4.2. The signal Intensity, i.e., the Telomere Length

The analysis of the signal intensity, i.e., the telomere length displayed a different profile between both groups. The total intensity variation of telomeres between both surgeries in group-2 patients was highly significant (*p* = 0.002) but it was not so significant in group-1 patients (*p* = 0.08).

#### 2.4.3. The Number of Telomere Aggregates

We analyzed the number of telomere aggregates, translating telomere fusions or clusters of telomeres measured by Teloview^®^, that are found in close proximity and cannot be further resolved as separate entities by microscopy at an optical resolution limit of 200 nm. This analysis was again carried out at first and second surgery in both groups. We found a significant difference in the total number of telomeric aggregates between both surgeries in group-1 patients (*p* < 0.001) but not in group 2-patients (*p* = 0.09).

#### 2.4.4. Nuclear Volume i.e., Volume of Each Cell

The assessment of the nuclear volume of the cell displayed a highly significant variation between both surgeries in group-1 patients (*p* < 0.0001), whereas the difference was not significant in group-2 patients (*p* = 0.07).

#### 2.4.5. The *a/c* Ratio and Telomere Distributions per Nuclear Volume

The *a*/*c* ratios is determined by representing the nuclear space occupied by the telomeres as an ovoid, with two main axes, *a* and *b*, that are equal in length, and a third axis, *c*, that has a different length [42]. This distribution of telomeres in the three-dimensional space of the nucleus varies with cell cycle; as the specific stages of the cell cycle (G0/G1, S, and G2) phases have characteristic *a*/*c* ratios, one can determine where they reside in the cell cycle [42]. The *a*/*c* ratio is a mean of defining progression through cell cycle in interphase cells [41,42]. Finally, the telomere distributions per nuclear volume i.e., the distance of each telomere from the nuclear center versus the periphery were measured. These three parameters (nuclear volume, the *a*/*c* ratio and the distribution of the telomeres per nuclear volume) allow for the characterization of cell cycle distribution, similar to Ki67 [41,45,46] (Table 1), cell size and overall distribution of telomeres within the 3D nuclear space. For both groups, these parameters were not significantly different between both surgeries (*p =* 1; *p =* 0.10; and *p =* 0.95) except for *a*/*c* ratio in group-2 patients which is significantly different between both surgeries (*p =* 0.02).

All these results suggest a significant difference between 3D telomere parameters when comparing both surgeries in group-1 patients. However, this is not the case in group-2 patients, in which most of the parameters are similar between both surgeries.

### 2.5. Clinical Significance of Both Groups

After the analyses of the 3D telomere profiles, the samples were decoded (unblinded) and we compared the time to progression or recurrence (time between both surgeries) between both groups. We observed that tumor recurrence is significantly different for both groups:

− group 1 presented a mean of 231.375 ± 117.42 days and a median TTP of 239 days that we coined “short term–recurrence” (Figure 2; Table 2).
− group 2, on the other hand, depicted a mean of 960.666 ± 86.19 days and a median TTP of 930 days, thereby considered “long–term recurrence” (Figure 3; Table 2).

Thus, 3D telomere analyses appear able to predict recurrence intervals in oligodendroglial tumors. Indeed, both short- and long-term recurrence groups are characterized by significantly different and identical 3D telomere profiles, respectively.

### 2.6. Time to Progression (TTP) and Overall Survival (OS) in Both Groups

**Figure 4:**
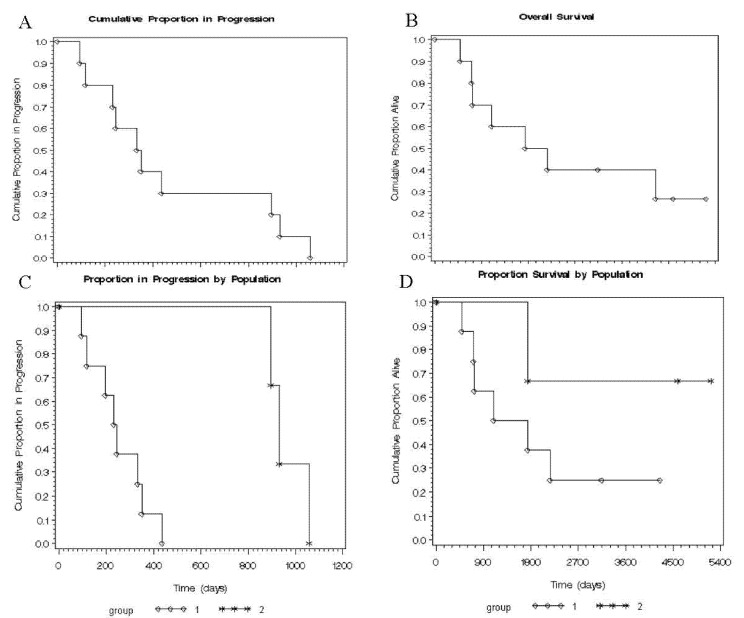
Kaplan-Meier curves depicting the time to progression (**A**) and overall survival (**B**) for all population (combination of group 1 and group 2); TTP (days between first surgery to second surgery for each patient of each group) (**C**) (*p* = 0.0078) and OS (days between diagnosis and end-point for each patient of each group) (**D**) (*p* = 0.096) of both defined groups by 3D telomere profiles.

In terms of clinical surrogates, we compared the time to progression (TTP) and overall survival (OS) in both groups. The OS and the TTP follow the general trend of disease progression in oligodendrogliomas, but OS between both groups was similar (*p =* 0.094). However, the Kaplan-Meier curves for TTP displayed a highly significant difference (*p =* 0.0078) between both groups as defined by 3D telomere profile of recurrence (Figure 4, Table 1). Thereby, not only do the 3D telomeres profiles predict recurrence delay in these diseases but they also predict TTP in oligodendrogliomas.

## 3. Discussion

To improve the quality of life and survival time of patients, there is a real need to investigate prognostic factors of oligodendrogliomas, in the goal to identify and treat high risk patient group with an aggressive regime to avoid or delay disease recurrence [5]. Indeed, recurrence is a clinical feature of oligodendrogliomas over progression.

Since the first morphologic description of gliomas by Bailey and Cushing in 1926, three WHO classifications (1979, 1993 and 2000) were established based mostly on morphologic and histological appearance of the glial cells [4,7,8,9,47,48,49,50]. Subsequently, a fourth WHO classification of brain tumors was published in 2007, which was updated in 2016 and was mostly based on molecular and genetic findings [25,50,51,52,53]. The WHO 2016 defined each gliomas entity by genetic and molecular characteristics among on top of the histology. Thus, this revised version includes *IDH* mutations and 1p/19q codeletion as central biomarkers for the diagnosis of diffuse gliomas [26,50]. Furthermore, the “integrated diagnosis” for infiltrating gliomas requires assessment of the tumor for *IDH* mutations and 1p/19q codeletion [53]. This updated WHO 2016 classification, which includes molecular markers, demonstrates the heterogeneity of different malignant brain tumors and the difficulty of classifying these tumors using histology alone [26,50]. However, this new classification has limitations to characterize these heterogeneous tumors. New biomarkers for diagnostic, prognostic and response to therapy are a major concern for the management of patients with gliomas [26]. Furthermore, the tumor heterogeneity is a key component of causal recurrence in these diseases and reflects genomic instability in different cellular clonal evolution. One of the driving force of genomic instability is telomere dysfunction and erosion [54]. Indeed, the growing list of options for cytogenetic analysis has improved the understanding of chromosomal changes in disease initiation, progression, and response to treatment. Oligodendrogliomas are now molecularly defined by the simultaneous presence of *IDH* mutations and codeletion of chromosomal arms 1p and 19q. Furthermore, 1p/19q codeletion has predictive value in terms of response to specific chemotherapy regimens [4]. However, the disease progression and the underlying mechanisms of recurrence remain unknown.

In prior studies, we have gained a mechanistic understanding of 3D telomeric organization in several tumors [39,43,44,54,55,56,57,58,59], and we deciphered the mechanistic transition of mononuclear Hodgkin cells to multinuclear Reed-Sternberg cells [58] and differentiated refractory and/or relapsing Hodgkin lymphoma patients from those who rapidly enter sustained remission [59]. We have also defined for the first time distinct telomeric profiles specific to patients with myelodysplastic syndromes (MDS) or acute myeloid leukemia (AML), and have suggested for the first time a chronological and evolutionary process of telomere dysfunction from MDS to AML [44]. Furthermore, we have previously identified specific 3D telomeric signatures for glioblastoma patient with short-term (92 days), intermediate (264 days) and long-term median survival (591 days) [41]. This subsequently allowed us to stratify glioblastoma patients into three distinct and highly predictive prognostic categories. These data enabled us to propose the nuclear telomere architecture as a novel biomarker of glioblastoma [41].

Prompted by these above data, we applied 3D telomere architecture on oligodendrogliomas before and after relapse to gain insight into mechanisms of recurrence. This was combined to a study of 1p/19q deletion status for each patient using our procedure [3]. Interestingly, in the current study, we found two groups of patients dichotomized by their 3D telomere profiles at diagnosis and after recurrence. Group 1 included eight patients who displayed significantly different 3D telomere profiles between first and second surgeries (*p* < 0.0001). These patients were categorized as having short-term recurrence as their recurrence occurred at a mean of 231.375 ± 117.42 days and a median TTP of 239 days. In the second group, three patients displayed identical 3D telomere profiles between both surgeries (*p* > 0.05) suggesting that tumor genotype was not significantly altered. These patients were categorized as having long-term recurrence as their recurrence occurred at a mean of 960.666 ± 86.19 days and a median TTP of 930 days. In addition, the value of 1p/19q deletion as a biomarker of chemosensitivity response of oligodendrogliomas, has already been established [20] and is further supported here by the overall survival of our patient-cohort and the lack of difference in both groups of patients considering both deletion.

The 1p/19q deletions were assessed by classical FISH using molecular probes mapping 1p36.2/1q25.2 and 19p13.2/19q13.3 on chromosomes 1 and 19, respectively [3,4,60]. However, microdeletions in 1p and 19q may result in false positive 1p/19q codeletion results as tested by FISH in the absence of whole arm deletions [53,61]. Indeed, FISH may give false positive results on FISH analysis [3,62,63], and appeared to be insufficient to fully distinguish oligodendrogliomas from other brain tumors (usually glioblastoma, and glioblastoma with oligodendroglial component) that harbor focal deletions of 1p and 19q [3,62,63]. For this reason, in the WHO 2016 classification, it is suggested to use molecular testing by a method that assess whole-arm chromosomal loss, such as molecular inversion probe array, single nucleotide polymorphism, chromosomal microarrays or next-generation sequencing with copy number analysis [64]. Finally, it is shown that 30 to 40% of oligodendrogliomas have an intact 1p and 19q chromosome arms, but follow a worse prognosis and were considered to be astrocytic in nature [14]. Other common genetic alterations recognized in these tumors include mutations of the *TP53* gene found in 30% of cases [65]. These alterations seem to be mutually exclusive, i.e., tumors with 1p-/19q- do not usually exhibit mutations of the *TP53* gene and vice-versa, implying a clonality of these neoplasms [65]. In addition *p16* gene deletions are common progression-associated alterations while 10q deletions and EGFR amplifications are unusual [66]. More recently, the presence of polysomy in anaplastic oligodendrogliomas with 1p-/19q- has been described as a marker of earlier recurrence [67]. Polysomy seems to be more frequent in recurrent and high-grade tumors [66,67]. The 1p/19q deletions is useless for prediction of time to recurrence in oligodendrogliomas as done by 3D telomere profiling.

Recently, high-throughput sequencing efforts of oligodendrogliomas identified different anomalies [68,69,70]. Amongst others, recurrent somatic mutations and insertions/deletions in *CIC,* a gene on chromosome 19q13.2 were described. Mutations in *FUBP1*, *IDH1 and IDH2* were found suggesting a functional interaction between *CIC* mutation, *IDH1*/*2*, and 1p/19q deletion [67,68,69,70]. A four-microRNA signature was shown able to identify patients with lower-grade gliomas under high risk of mortality [70]. A low serum level of microRNA-376 was identified as an independent factor predicting poor outcome of glioma patients [71]. A mutation of BRAF, V600E, was associated with an improved overall survival among glioma patients [72,73,74]. None of these markers are specific to oligodendrogliomas despite being universal features of this disease.

Thus, there is a strong need for further molecular investigations including some combination of *IDH*, 1p/19q codeletion and the tumor’s telomere maintenance mechanism, defined by alterations in either *TERT* or *ATRX* associated to 3D telomere profiling quantifying the level of genomic instability and tumor heterogeneity [27,28,53]. Indeed, *TERT* promoter mutations and *ATRX* alterations have been shown to be associated with prognosis and reinforce our preliminary data of 3D telomere profiling [53]. Therefore, nuclear telomere architecture as in glioblastomas [41,54] might be a valuable biomarker to monitor disease progression as well as predict time to recurrence in oligodendrogliomas. Finally, telomere dysfunction might be a driving event to recurrence in oligodendrogliomas.

Although this is a pilot study, our data suggest that 3D telomere profiling may assist in the identification of short- or long-time to progression in oligodendrogliomas. Our finding should be validated in independent patient cohorts with a higher number of patients and in combination with IDH, TERT, and ATRX assessments to classify oligodendrogliomas according to WHO 2016 criteria combined with the analyses of telomerase versus alternative length of telomeres (ALT) activity for the maintenance of telomere length. Thus, the 3D nuclear telomere organization preceding genomic instability and predicting recurrence, will be a biomarker helping to define new algorithm for accurate diagnosis, better treatment and follow-up of oligodendrogliomas.

## 4. Materials and Methods

### 4.1. Patients

This study received approval by the research ethics board on human studies (11-088/2012-09-10) at the Centre Hospitalier Universitaire de Sherbrooke (CHUS). Patients undergoing or having undergone surgery for an initially diagnosed oligodendroglial brain tumor at the CHUS were enrolled in this study after informed consent. Only post-operative oligodendroglial histology patients that underwent at least two surgical procedures, and thus presented at least one episode of tumor recurrence, were considered in the present analysis.

### 4.2. Samples

Fresh surgical biopsies were obtained and collected immediately after resection in the operating room, prospectively. Immediately after collection, touch preparation smear slides (TP slides) were prepared by smearing a core biopsy onto a glass slide [3]. The TP slides were then fixed using fresh fixative (Carnoy: 3 vol methanol/1 vol glacial acetic acid), air-dried in a chemical hood, and stored at -20˚C until needed for FISH and Q-FISH.

### 4.3. Histopathological Diagnosis

Tumors were reviewed and classified according to the 2007 World Health Organization (WHO) classification [2]. After surgical removal, fragments of tumor were fixed in 10% formaldehyde and embedded in paraffin. Three-micrometer sections were stained with hematoxylin and eosin and submitted to immunohistochemical reactions for the detection of glial fibrillary acidic protein (GFAP), neurofilaments, and synaptophysin. Additional sections were submitted for Ki-67 antibody staining (all antibodies from DAKO, Carpinteria, CA, USA) to evaluate the proliferative index (Table 1). Tumor vascularization and degree of microvascular proliferation were evaluated with CD31 antibody (DAKO, Carpinteria, USA). Diagnosis and grading were established according to WHO criteria [2].

### 4.4. Molecular Cytogenetic Analysis

Fluorescence in situ hybridization (FISH) was performed using Vysis LSI 1p36/1q25 and LSI 19q13/19p13 dual-color probe sets (Abbott Molecular, Des Plaines, IL, USA) according to the manufacturer’s protocol and our previously described procedure [3] on TP smear slides. For chromosome 1, the LSI 1p36 Spectrum Orange probe and the control part LSI 1q25 Spectrum Green probe map 435 kb and 618 kb on the 1p36 and 1q25, respectively. For chromosome 19, the LSI 19q13 Spectrum Orange probe and the control part LSI 19p13 Spectrum Green probe map 380 kb and 502 kb on the 19q13 and 19p13, respectively. Indeed, FISH targeting 1p36/1p21 and 19q13/19p13 regions via fluorophore- labelled DNA probes [66], was used as standard protocol to detect 1p/19q status in most hospitals [67].

#### 4.4.1. FISH Analysis

An average of 200 nuclei per slide were scored in the FISH analysis. Only the non-overlapping, morphologically well-preserved nuclei were included in the analysis for TP slides. However, in TP slides, almost all nuclei were non-overlapping allowing the analysis [3]. For each cell, the ratio between the paired probes on chromosomes 1 (LSI 1p36 Spectrum Orange probe and the control part LSI 1q25 Spectrum Green probe) and 19 (LSI 19q13 Spectrum Orange probe and the control part LSI 19p13 Spectrum Green probe) was analyzed. The green signal in the used probes served as a control, and the deletion per nucleus showed that the number of green signals was higher than the number of red signals (target signals). A case was considered deleted with 1p, 19q, or both when the scored nuclei displayed an imbalance between the green and red signals following our procedure as previously described (Figure 1) [3].

#### 4.4.2. Image Acquisition for FISH with 1p/19q Probes

Each slide was examined using an Olympus BX61 microscope equipped with appropriate filters at a 1 × 1000 magnification. The pictures of selected cells were taken using a Compulog IMAC-CCD S30 video camera module (MetaSystems Inc, Altlussheim, Germany, Belmont, MA, USA) and were analyzed using the in situ imaging system (ISIS 2) software version 2.5 (MetaSystems Inc, Altlussheim, Germany; Belmont, MA, USA).

### 4.5. Quantitative—Fluorescent in Situ Hybridization (Q-FISH) Protocol for 3D Analysis

The TP slides were thawed for 1 h at room temperature (RT). The procedure was performed as described previously [41,42]. Briefly, slides were incubated in 3.7% formaldehyde/phosphate-buffered saline (PBS; pH 7.4) for 20 min and washed 3× in 1× PBS for 5 min each. Slides were incubated in 0.5% Triton X-100 for 3 min followed by an incubation in 20% glycerol for 1 h, and 3D-preserved by three repeated cycles of glycerol/liquid nitrogen treatment and washed twice in 1xPBS for 5 min each followed by a 5 min incubation in 0.1 N HCl. Prior to fixation in 70% formamide/2× SSC at pH 7.0 for 1 h, slides were washed twice for 5 min in 1× PBS. Immediately after fixation, 8 µL of PNA telomeric probe (Dako; Glostrup, Denmark) was added to the slide. For denaturation of the nuclear DNA and the probe, the slides were incubated at 80 °C for 3 min followed by hybridization at 30 °C for 2 h using a Hybrite^TM^ (Vysis; Abbott Diagnostics, Des Plains, IL, USA). The slides were washed twice for 15 min each in 70% formamide/10 mM Tris pH 7.4 followed by washing for one min in 1× PBS at room-temperature (RT) while shaking and in 0.1× SSC at 55 °C for 5 min while shaking. Slides were washed in 2× SSC/0.05% Tween 20 twice for 5 min each at RT while shaking, after which they were counterstained with 4′,6-diamino-2-phenylindole (DAPI) (0.1 µL/mL). Excess DAPI was removed with deionized distilled water prior to dehydration in ethanol at 70%, 90%, and 100% for 2 min each. The slides were then air-dried, and cover slipped with Vectashield (Vector Laboratories, Burlington, ON, Canada) for analysis.

### 4.6. Image Acquisition and 3D Image Analysis Using TeloView^®^

Imaging data from all patient oligodendroglioma tissues were obtained by standard acquisition method [42]. We performed 3D image analysis on 30 interphase nuclei per slide using an AxioImager Z1 microscope (Carl Zeiss Canada Ltd., Toronto, ON, Canada) and an AxioCam HRm charge-coupled device (Carl Zeiss Canada Ltd., Toronto, ON, Canada) [43]. A 63-x oil objective lens (Carl Zeiss Canada Ltd., Toronto, ON, Canada) was used at acquisition times of 366 milliseconds (ms) for Cy3 (telomeres) and 109 ms for DAPI (nuclei). Sixty z-stacks were acquired at a sampling distance of xy: 107 nm and z: 200 nm for each slice of the stack. Axiovision 4.6 software (Carl Zeiss Canada Ltd., Toronto, ON, Canada) and a constrained iterative algorithm [75] were used for deconvolution. Deconvolved images were converted into TIFF files and exported for 3D-analysis using the TeloView^®^ software platform (Telo Genomics Corp., Toronto, ON, Canada) [42].

### 4.7. Data Presentation and Statistical Analysis

The TeloView^®^ software platform [41,42,43,44] computes six parameters that constitute the 3D telomere profile for each sample. These parameters include telomere length (signal intensity), telomere numbers (number of signals), telomere aggregates, the nuclear volume, the a/c ratios and the telomere distribution in the nucleus. The software generates for each sample three types of histogram and calculates the percentage of cells having telomere aggregates, the mean number of signals, and the mean number of aggregates per cell [41,42,43,44]. The histogram data from the different surgery samples of each patient were combined into a single chart for comparison (see Figure 2E and Figure 3E; and Supplemental Figures S1–S8).

### 4.8. Statistical Analyses and Overall Survival

For each patient, samples from both surgeries at diagnosis and recurrence were analyzed blindly and subsequently compared based on their 3D telomeric profiles. Because patient 4 (P4), underwent four surgical procedures, we analyzed all four surgeries. The telomeric parameters (number, length, telomere aggregates, nuclear volumes, and *a*/*c* ratio) were compared using a randomized block analysis of variance. Distribution of telomere intensities was compared by chi-square analysis. Multiple pairwise comparisons using a least square mean tests followed a significant omnibus overall effect. Two groups of patients’ cell parameter averages were analyzed over both surgeries with nested factorial analysis of variance taking both patient and cellular variations into account. Telomere intensity groups were compared between the two patient groups using Breslow-Day test for homogeneity of odds ratios. Significance level was set at α = 0.05.

Kaplan-Meier curves were estimated for survival and time to progression. Survival time was measured from date of diagnosis and censored at the same time of follow-up fixed arbitrary at the date of data analysis or patient death. Survival curves were compared with the log-rank tests. Significance level was set at α = 0.05.

## 5. Conclusions

Our results define for the first time a link between nuclear telomeric architecture and telomere dysfunction with oligodendrogliomas recurrence. Indeed, 3D telomere analyses allow a stratification of oligodendrogliomas into short-term and long-term recurrence groups independently of the 1p/19q deletion status. Short-term recurrence patients were characterized by significantly different 3D telomere profiles whereas long-term recurrence patients displayed identical 3D telomere profiles. Thus, 3D telomere profiles were able to predict TTP. This reinforces the potential of nuclear telomere organization as a strong biomarker in oligodendrogliomas.

## 6. Patents

Title; “Method of monitoring genomic instability using 3D microscopy and analysis” [ID Canada:2,515,792; ID USA:7,801,682; ID France: EP4713499.4; EP1594990; ID Germany: EP04713499.4; 048302.8; ID Spain: EP04713499.4; ES2567199; ID United Kingdom: EP04713499.4; EP1594990].

## Figures and Tables

**Table 1:** Clinical data and FISH of 1p/19q deletion.

ID and Exponent 1 or 0		Age	Sex	KI67	TTP (day)	OS (day)	Surgery	Diagnostic and Clinical Evolution	1p/19q Status
^1^ P1		40	F	5%	894	5212	1	Oligodendroglioma Mid aggressive	1p-/19q-
							2	Oligodendroglioma	1p-/19q-
^1^ P2		40	F	++	350	2172	1	Oligodendroglioma Mid aggressive	1p-/19q-
							2	Oligodendroglioma	1p-/19q-
^1^ P3		42	M	15%	95	721	1	Oligodendroglioma aggressive	1p-
							2	Oligodendroglioma	1p-
^1^ P4	P4a	29	M	++	930	1748	1	Oligodendroglioma indolent	1p-/19q- Polysomic
							2	Oligodendroglioma	1p-/19q- Polysomic
	P4b			++	197		3	Oligodendroglioma	19q-
							4	Oligoastrocytoma	19q-
^1^ P5		33	F	>40%	335	4242	1	Oligodendroglioma Mid aggressive	1p-/19q- Polysomic
							2	Oligodendroglioma	1p-/19q- Polysomic
^0^ P6		55	F	5%	1058	4582	1	Oligodendroglioma indolent	1p-/19q-
							2	Oligodendroglioma	1p-/19q-
^0^ P7		37	M	>25%	437	3141	1	Oligodendroglioma indolent	1p-/19q-
							2	Anaplastic oligodendroglioma	1p-/19q-
^1^ P8		47	F	Hetero-geneous till 60%	246	500	1	Anaplastic oligodendroglioma Invasive	19q- Polysomic
							2	Anaplastic oligodendroglioma	19q- Polysomic
^1^ P9		79	M	>30%	232	1103	1	Anaplastic oligodendroglioma Aggressive	1p-/19q- Polysomic
							2	Anaplastic oligodendroglioma	1p- Polysomic
^1^ P10		46	F	Nd	119	735	1	Oligodendroglioma aggressive	1p- Polysomic
							2	Oligodendroglioma	1p- Polysomic

^0^: alive; ^1^: dead; TTP: days between first surgery (diagnosis) to second surgery; OS: days between first surgery (diagnosis) to end point. ++: mild or moderate expression

**Table 2:** Comparison of telomeric profile parameters per cell between the two defined groups.

Group	Level of Surgery	Total Number of Signals		Total Number of Aggregates		Total Intensity		Average Intensity of All Signals		Nuclear Volume		*a*/*c* Ratio		Telomere per Nuclear Volume	
		Mean	Std Dev	Mean	Std Dev	Mean	Std Dev	Mean	Std Dev	Mean	Std Dev	Mean	Std Dev	Mean	Std Dev
1	1a	29.8444816	15.5864981	3.23411371	2.64075267	569,912.151	281,821.009	20,387.6865	8092.16645	954,885.37	527,813.807	9.527	5.8	0.03802044	0.02309804
	2a	33.5899160	18.0550011	3.79327731	2.90812548	544,668.245	290,813.157	16,772.3811	4445.41517	1,131,519.38	961,970.627	12.749	45.4	0.27817647	4.15627785
	*p* value 1a-2a	<0.0001		<0.0001		0.0804		<0.0001		<0.0001		1.0000		0.1053	
2	1b	29.5312500	19.7783065	3.28125000	3.27593066	427,011.450	190,557.547	16,499.5065	5437.76924	745,605.69	446,145.342	827,766.671	10,470,359.1	0.06224833	0.06443754
	2b	32.4500000	17.6534779	3.72500000	2.89425803	528,013.631	219,867.712	18,502.6929	7327.63749	865,677.43	470,170.711	7.255	4.4	0.04492112	0.02969461
	*p* value 1b-2b	0.0365		0.0929		0.0002		0.0020		0.0799		0.0299		0.9524	
Group 1 versus group 2 with the different surgeries	*p* value 1a-1b	0.0518		0.0803		<0.0001		<0.0001		<0.0001		0.0540		0.6117	
	*p* value 1a-2b	<0.0001		0.0002		0.0236		<0.0001		<0.0001		0.5573		0.5642	
	*p* value 2a-1b	0.2070		0.4434		<0.0001		0.0017		<0.0001		0.0542		0.1398	
	*p* value 2a -2b	0.2471		0.2394		0.2244		0.6714		<0.0001		0.5577		0.1222	

1a: group 1 first surgery; 2a: group 1 second surgery; 1b: group 2 first surgery; 2b: group 2 second surgery.

## Supplementary Materials

Supplementary materials can be found at https://www.mdpi.com/1422-0067/21/22/8539/s1, Figures S1–S8: Title: Combined 3D telomere profiles displaying the distribution of the number of signals (total number of telomeres) versus their intensities (telomere length) at both surgeries, respectively; from patients P2, P3, P4a, P4b, P5, P6, P7, P8, and P10; Table S1: Comparison between telomeric profile parameters per cell at first surgery and recurrence per each patient.

## Abbreviations

3D	three-dimensional
AML	acute myeloid leukemia
CHUS	Centre Hospitalier Universitaire de Sherbrooke
CIN	chromosomal instability
DAPI	4′,6-diamidino-2-phenylindole
FISH	fluorescence in situ hybridization
GBM	Glioblastoma
MDS	myelodysplastic syndromes
OS	overall survival
PNA	peptide nuclei acid
Q-FISH	Quantitative-Fluorescent in situ hybridization
RT	room temperature
TA	telomere aggregates
TP	touch preparation
TTP	time to progression
WHO	World Health Organization
