# Supplementary material for: Three-Dimensional Nuclear Telomere Profiling as a Biomarker for Recurrence in Oligodendrogliomas: A Pilot Study
Source: Int J Mol Sci. 2020 Nov 12;21(22):8539. doi: 10.3390/ijms21228539 (PMC7696868; doi:10.3390/ijms21228539)
Supplement: Supplementary file 1 [file ijms-21-08539-s001.pdf]

International Journal of
Molecular Sciences

MDPI

Supplemental Materials

Figure S1–S8: Title: Combined 3D telomere profiles displaying the distribution of the number of signals (total number of telomeres) versus their intensities (telomere length) at both surgeries, respectively; from patients P2, P3, P4a, P4b, P5, P6, P7, P8, and P10

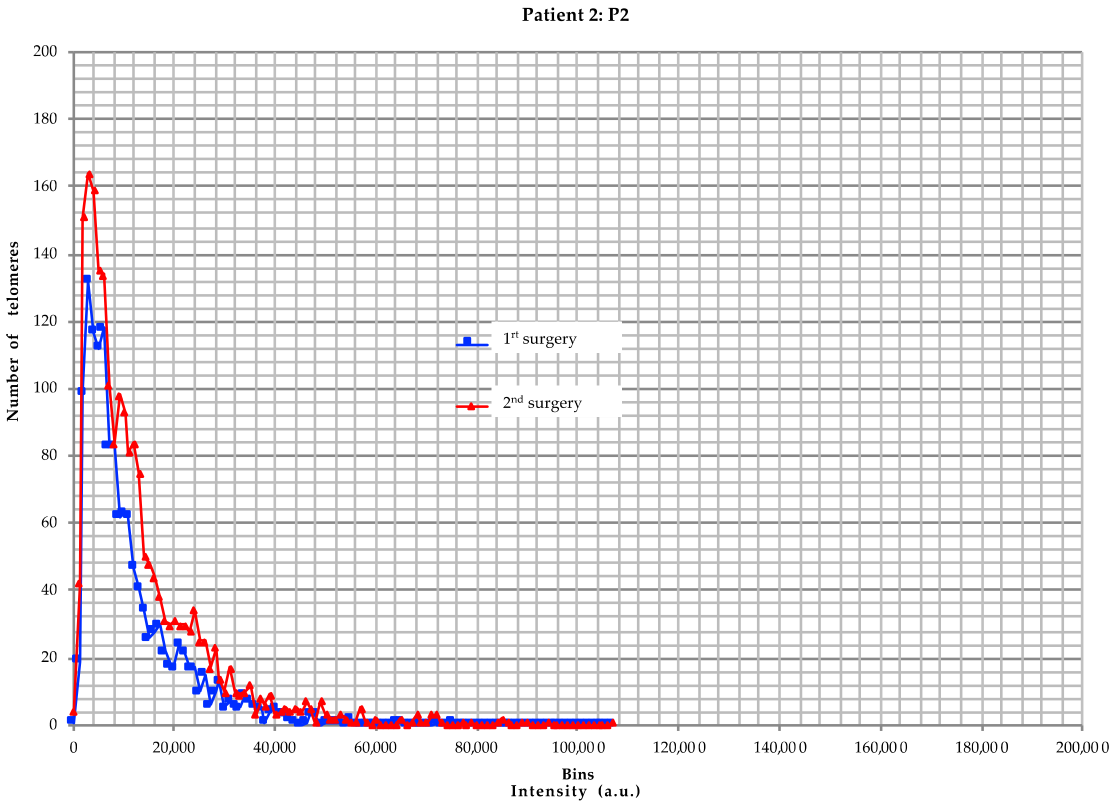

Figure S1: Combined 3D telomere profiles displaying the distribution of the number of signals (total number of telomeres) versus their intensities (telomere length) at both surgeries, respectively for patient P2 with short-term recurrence (TTP = 350 days and OS = 2172 days**).**

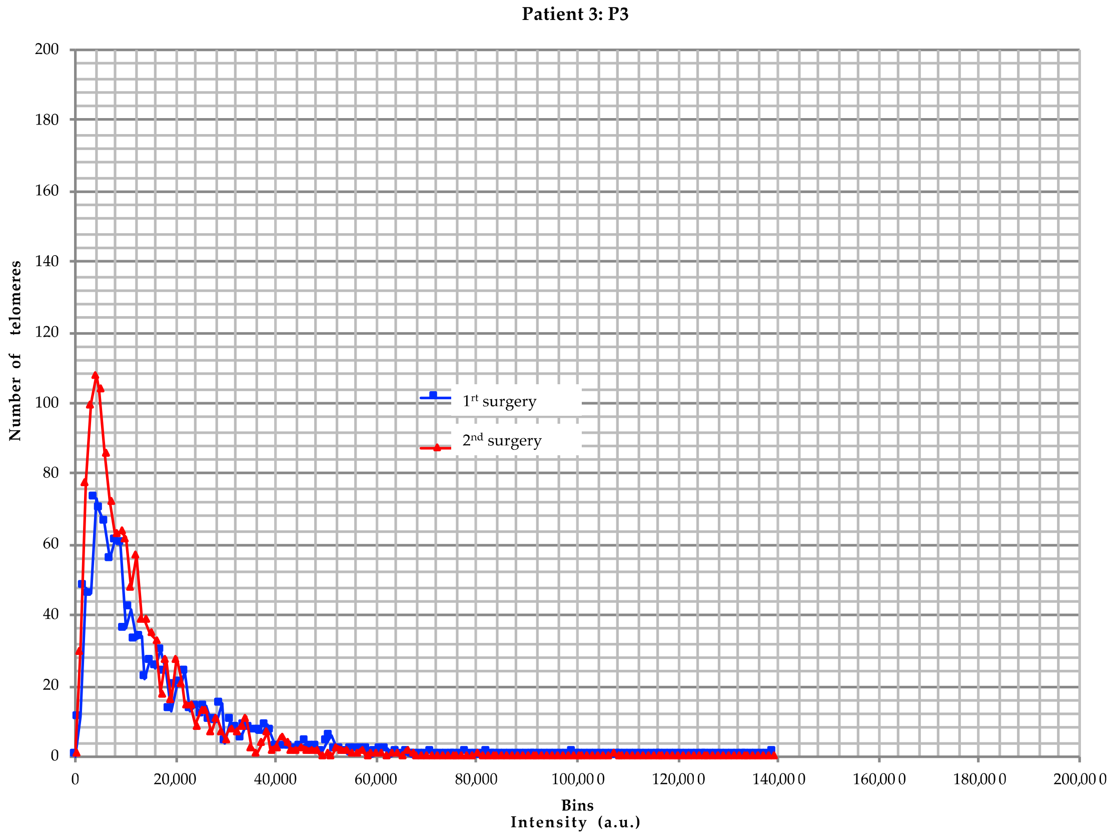

Figure S2: Combined 3D telomere profiles displaying the distribution of the number of signals (total number of telomeres) versus their intensities (telomere length) at both surgeries, respectively for patient P3 with short-term recurrence (TTP = 95 days and OS = 721 days).

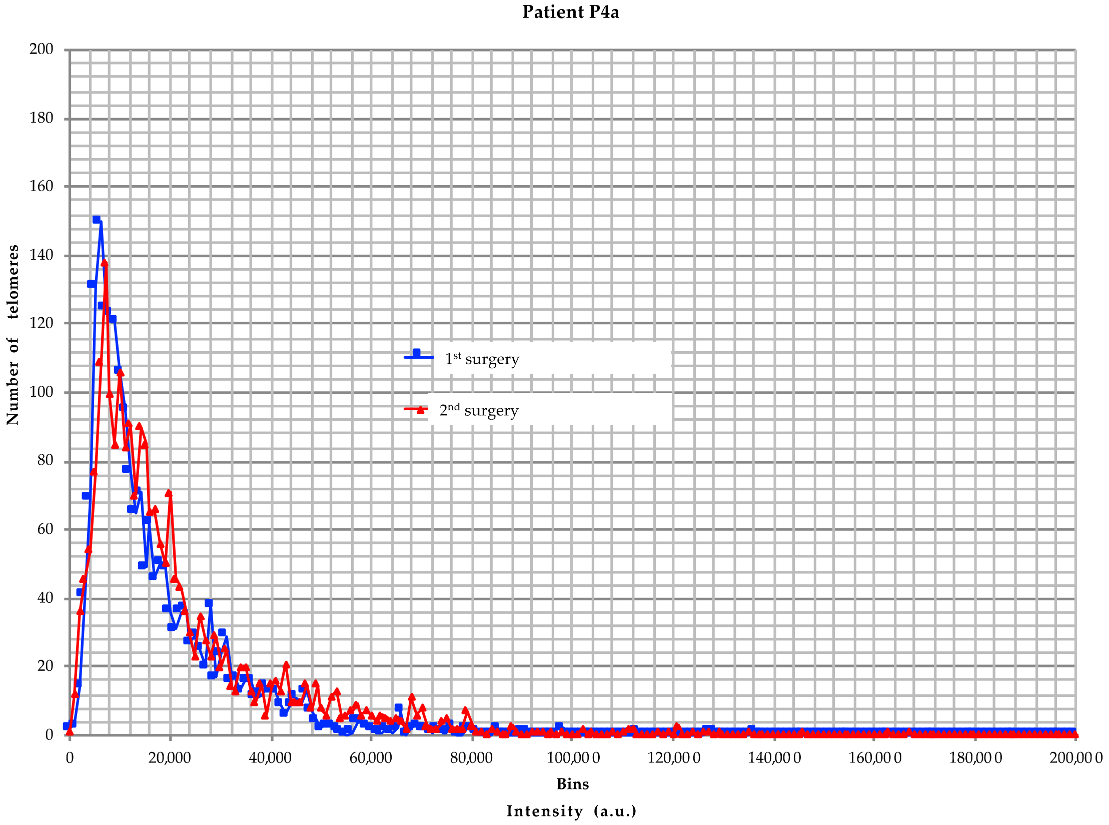

Figure S3-1: Combined 3D telomere profiles displaying the distribution of the number of signals (total number of telomeres) versus their intensities (telomere length) at surgery 1 and 2, respectively for patient P4 (P4a) with long-term recurrence (TTP = 930 days**).**

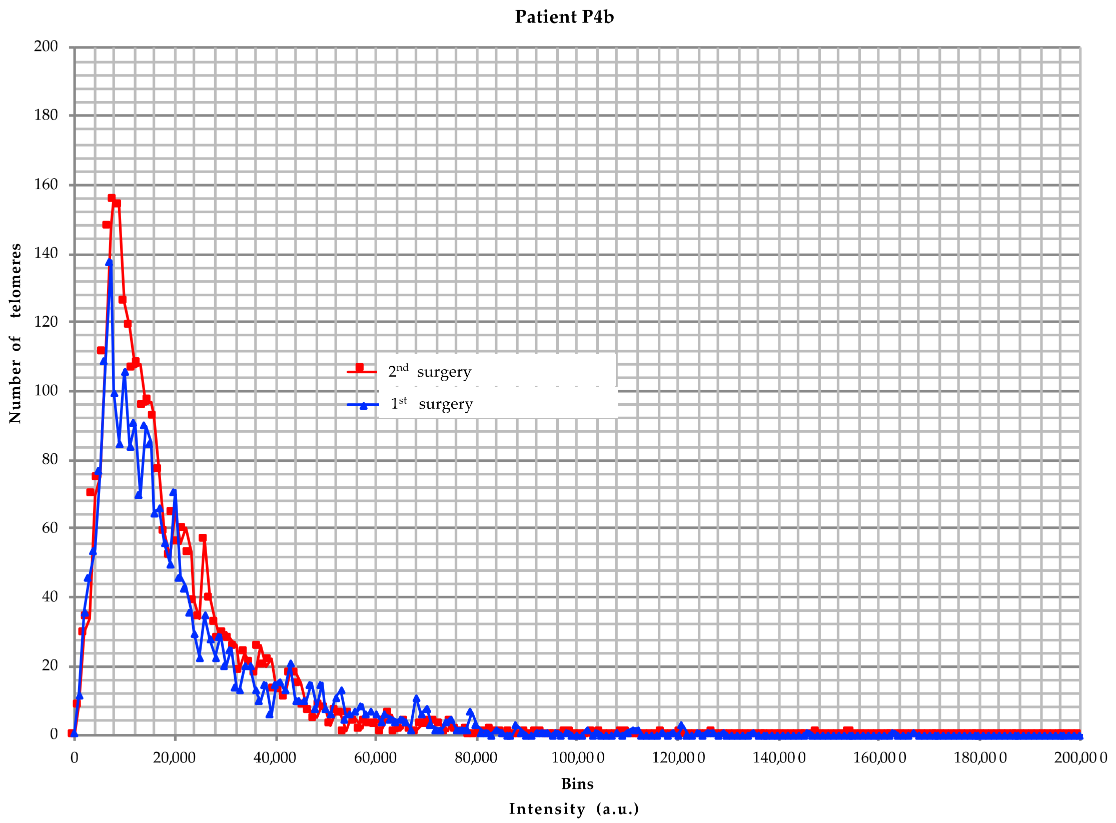

Figure S3-2: Combined 3D telomere profiles displaying the distribution of the number of signals (total number of telomeres) versus their intensities (telomere length) at surgery 3 and 4, respectively for patient P4 (P4b) with short term recurrence (TTP = 197 days and OS = 1748 days**)**.

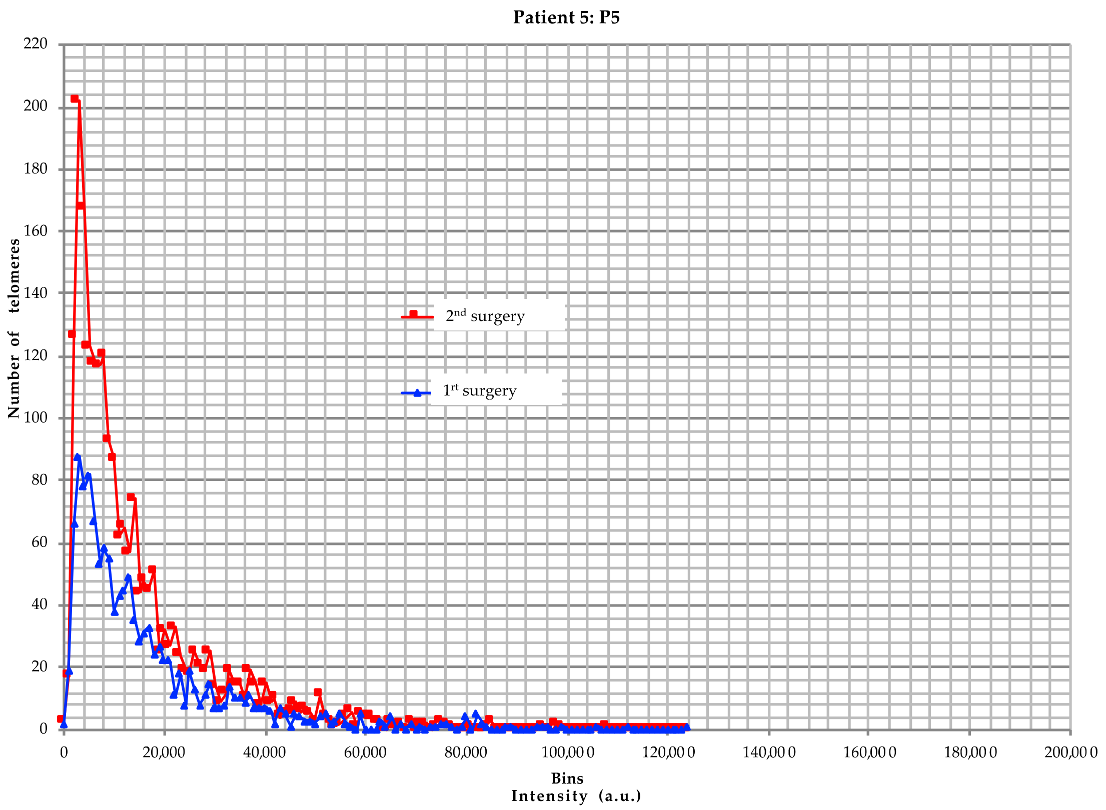

Figure S4: Combined 3D telomere profiles displaying the distribution of the number of signals (total number of telomeres) versus their intensities (telomere length) at both surgeries, respectively for patient P5 with short-term recurrence (TTP = 335 and OS = 4242 days)

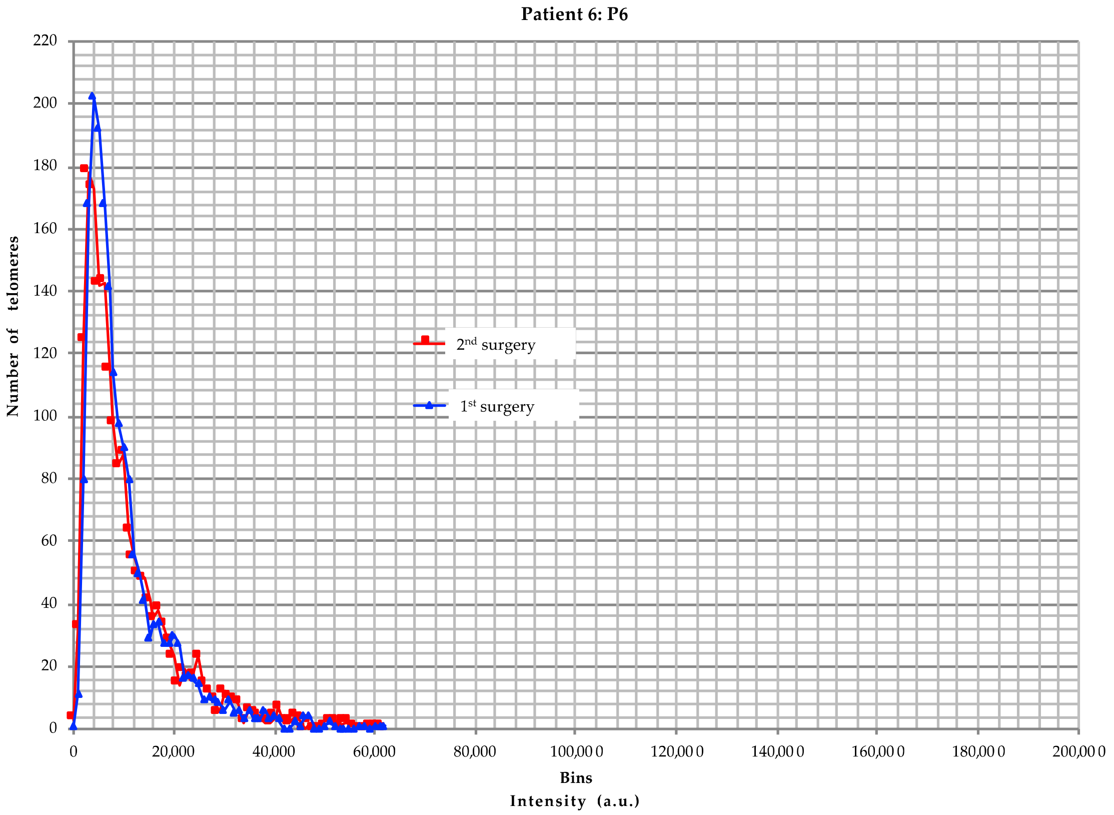

Figure S5: Combined 3D telomere profiles displaying the distribution of the number of signals (total number of telomeres) versus their intensities (telomere length) at both surgeries, respectively for patient P6 with long-term recurrence (TTP = 1058 days and OS = 4582 days)

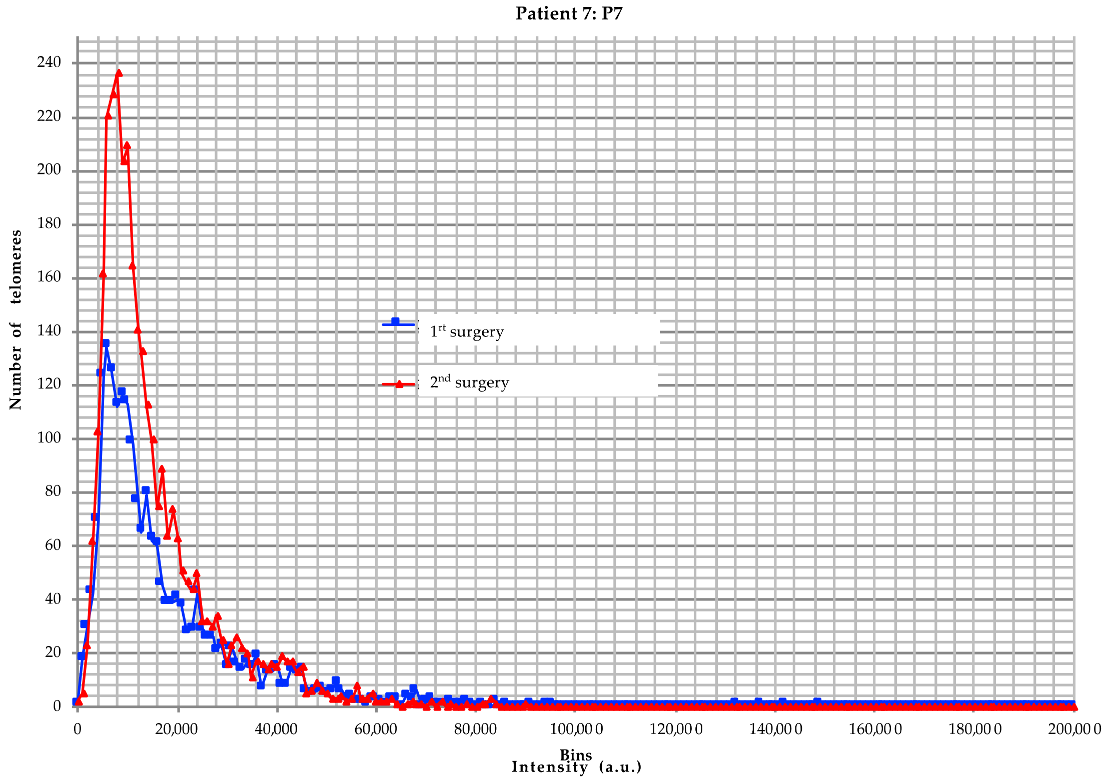

Figure S6: Combined 3D telomere profiles displaying the distribution of the number of signals (total number of telomeres) versus their intensities (telomere length) at both surgeries, respectively for patient P7 with short-term recurrence (TTP = 437 and OS = 3141 days)

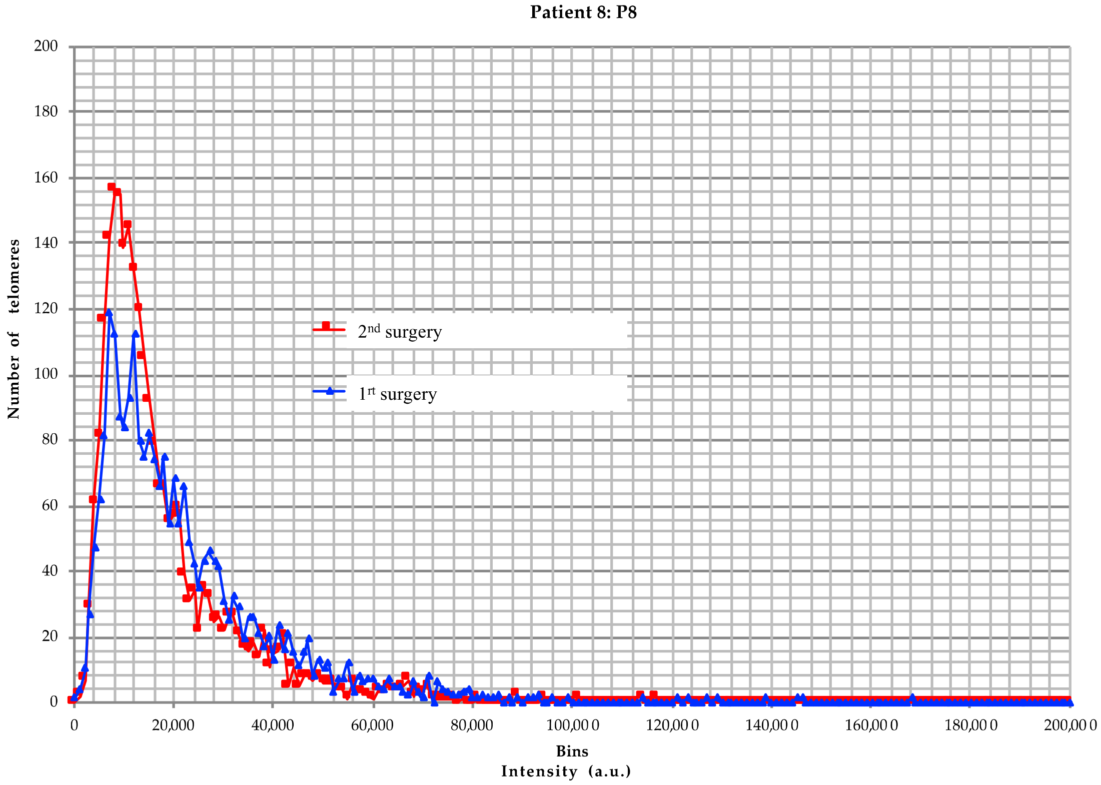

Figure S7: Combined 3D telomere profiles displaying the distribution of the number of signals (total number of telomeres) versus their intensities (telomere length) at both surgeries, respectively for patient P8 with short-term recurrence (TTP = 246 days and OS = 500 days)

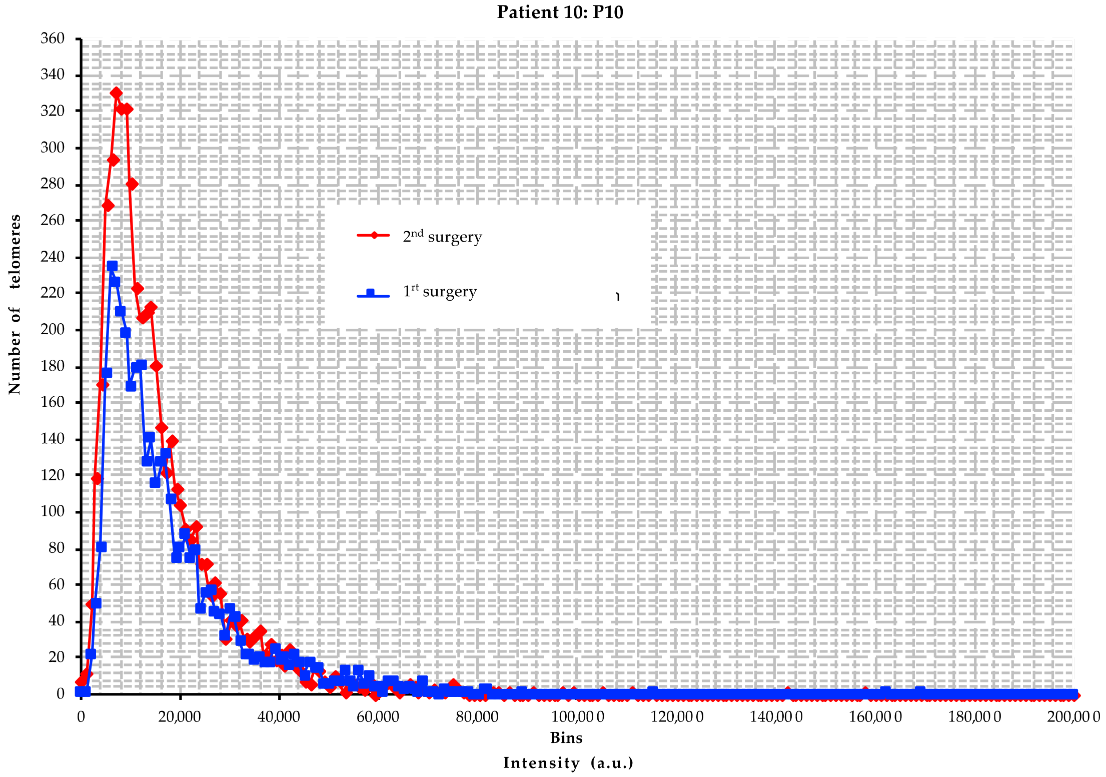

Figure S8: Combined 3D telomere profiles displaying the distribution of the number of signals (total number of telomeres) versus their intensities (telomere length) at both surgeries, respectively for patient P10 with short-term recurrence (TTP = 119 days and OS = 735 days)

Table S1: Comparison between telomeric profile parameters per cell at first surgery and recurrence per each patient

**Patient**	**Level of surgery**	**Totalnofsignals**		**Totalnofaggregates**		**Totalintensity**		**AvIntallsignals**		**Nuclearvolume**		**acratio**		**Telomere per nuclear volume**	
		**Mean**	**Std Dev**	**Mean**	**Std Dev**	**Mean**	**Std Dev**	**Mean**	**Std Dev**	**Mean**	**Std Dev**	**Mean**	**Std Dev**	**Mean**	**Std Dev**
P1	1	25.6666667	8.5956418	2.36666667	2.05918185	358551.10	115913.801	14199.4657	2444.1938	373671.13	148810.16	3.29	1.3	0.07587563	0.0309405
	2	36.3000000	8.1289182	3.96666667	2.20474931	453484.00	140191.851	12460.5273	2829.0921	972019.67	246068.92	4.35	1.9	0.03898138	0.0102518
	P value	0.0006		0.0087		0.1176		0.2349		<.0001		1.0000		0.9589	
P2	1	43.4545455	9.9375893	5.36363636	2.72509383	509486.12	142095.620	11820.0877	2823.5709	857304.76	672363.16	14.51	6.8	0.07424460	0.0431547
	2	69.6333333	17.4405064	7.70000000	3.66859928	947579.63	262010.421	13727.3887	2716.3457	3583927.27	1892694.79	10.41	4.7	1.67803312	9.0544901
	P value	<.0001		<.0001		<.0001		0.1824		<.0001		1.0000		0.0221	
P3	1	30.4857143	12.7056528	3.31428571	2.60961377	479234.77	194916.882	16358.2238	5378.1766	469016.06	197804.55	12.83	5.6	0.06770628	0.0202862
	2	37.8571429	21.0896446	4.48571429	3.93604331	482564.86	260277.395	13233.7060	3233.1756	391412.26	405606.97	7.40	9.3	0.14362152	0.0677502
	P value	0.0099		0.0378		0.9527		0.0212		0.5369		1.0000		0.9089	
P4	1	20.8000000	7.63299589	2.06000000	1.42715370	375758.110	127954.378	18915.4556	5245.02409	967354.54	418688.097	1324424.74	13244072.5	0.02450617	0.01163694
	2	22.8200000	8.79552228	2.57000000	1.87085569	506722.230	222179.793	22561.0009	6137.65197	894253.91	557762.655	8.08	5.0	0.03157469	0.01560654
	P value	P_{1-2}: 0.0727		P_{1-2}: 0.0395		P_{1-2}: <.0001		P_{1-2}: <.0001		P_{1-2}: 0.2796		P_{1-2}: 0.1581		P_{1-2}: 0.0004	
	3	26.5600000	8.76554319	3.19000000	1.93163457	520135.670	203616.405	19910.2126	5523.37587	641210.20	282388.439	5.65	2.6	0.04623971	0.01686107
	P value	P_{1-3}<.0001; P_{2-3}0.0009		P_{1-3}<.0001; P_{2-3}0.0124		P_{1-3}<.0001; P_{2-3}0.5881		P_{1-3}0.1900; P_{2-3}0.0005		P_{1-3}<.0001; P_{2-3}0.0002		P_{1-3}<.0.1581; P_{2-3}1.0000		P_{1-3}<.0001; P_{2-3}<.0001	
	4	22.5600000	6.29625510	2.68000000	1.71080937	398204.960	123737.368	17980.4619	4377.17820	1402065.67	587756.677	17.85	7.9	0.01900249	0.01038859
	P value	P_{1-4}0.1178; P_{2-4}0.8170; P_{3-4}0.0004		P_{1-4}0.0124; P_{2-4}0.6562; P_{3-4}0.0395		P_{1-4}0.3650; P_{2-4}<.0001; P_{3-4}<.0001		P_{1-4}0.2180; P_{2-4}<.0001; P_{3-4}0.0113		P_{1-4}<.0001; P_{2-4}<.0001; P_{3-4}<.0001		P_{1-4}0.1581; P_{2-4}1.0000; P_{3-4}1.0000		P_{1-4}0.0053; P_{2-4}<.0001; P_{3-4}<.0001	
P5	1	72.7666667	23.5630970	8.20000000	5.18884746	1116903.17	382680.970	15493.9587	2635.4038	1658196.77	592780.21	4.87	1.9	0.05132022	0.0310878
	2	42.4000000	12.6779744	5.53333333	2.31536596	728975.43	307871.044	17227.1018	4287.6478	1857352.00	792582.58	5.99	3.1	2.97789855	16.1640546
	P value	<.0001		<.0001		<.0001		0.2364		0.1424		1.0000		<.0001	
P6	1	62.5000000	21.6615821	8.26666667	3.99079400	666316.27	237938.941	10746.3837	1547.1771	378377.40	136253.54	3.15	1.3	0.17442824	0.0567762
	2	60.7000000	14.7581655	7.33333333	3.31489151	673514.60	218322.641	11017.1652	2294.3745	664080.27	181344.48	7.39	2.3	0.09534894	0.0248309
	P value	0.5592		0.1253		0.9055		0.8532		0.0354		1.0000		0.9121	
P7	1	20.9400000	7.5703035	2.31000000	1.66178072	394919.79	202847.524	19074.7775	7843.9860	910606.87	542192.02	8.51	6.0	0.03071166	0.0189959

	2	31.6900000	6.9887283	3.79000000	1.81071446	507602.54	136890.752	16152.7500	3074.6429	740300.30	232178.28	8.78	3.2	0.04562279	0.0140165
	P value	<.0001		<.0001		0.0007		0.0007		0.0221		1.0000		0.9697	
P8	1	24.1300000	6.3048082	2.36000000	1.62381270	553175.12	178958.768	23332.4983	6285.1285	965693.13	417750.76	10.26	4.2	0.02891512	0.0123079
	2	25.0700000	9.4732532	2.53000000	1.76071037	460400.26	205900.785	18259.8480	4898.8354	373972.76	202418.58	6.32	3.1	0.07794344	0.0321275
	P value	0.5777		0.6101		0.0053		0.0053		<.0001		1.0000		0.9006	
P9	1	23.2300000	7.9083832	2.13000000	1.52854985	613369.43	316858.437	27393.3880	11235.5051	915608.28	472247.61	11.42	7.7	0.02921808	0.0117040
	2	23.8600000	7.1421639	1.95000000	1.62912259	402310.16	158200.513	17287.1769	4811.8411	878065.05	360011.83	25.97	109.2	0.03189054	0.0164840
	P value	0.7090		0.5893		<.0001		<.0001		0.6135		1.0000		0.9946	
P10	1	36.7700000	11.9069244	3.96000000	2.36096197	655541.11	252227.853	17933.2939	4325.0578	1332570.84	403197.65	10.39	3.9	0.02868368	0.0090812
	2	49.8200000	21.2728214	6.08000000	3.26499648	800393.92	396503.460	16197.2864	4435.0919	1568758.44	671182.52	9.44	5.6	0.03364373	0.0121374
	P value	<.0001		<.0001		<.0001		0.0305		0.0015		1.0000		0.9899	
